# Fungal Viridionychia: Onychomycosis-Induced Chloronychia Caused by Candida parapsilosis-Associated Green Nail Discoloration

**DOI:** 10.7759/cureus.20335

**Published:** 2021-12-10

**Authors:** Parnia Forouzan, Philip R Cohen

**Affiliations:** 1 Medicine, McGovern Medical School, University of Texas Health Science Center at Houston, Houston, USA; 2 Dermatology, University of California Davis Medical Center, Sacramento, USA

**Keywords:** viridionychia, syndrome, onychomycosis, nail, green, goldman, fungal, fox, chloronychia, bacterial

## Abstract

Green nail syndrome is a form of chromonychia, discoloration of the nail plate, that describes fingernails or toenails that are green in appearance. Bacterial-associated green nail syndrome, referred to as chloronychia, is most common; however, fungal and polymicrobial etiologies have been reported. Two 70-year-old women presented with green discoloration of their nails for over five months; both women had prior unsuccessful treatments and were referred for further evaluation and treatment. The affected nails were biopsied and cultured. Bacterial cultures did not yield any organisms; however, fungal cultures grew *Candida parapsilosis *after four weeks. Both women were treated with a topical alcohol-based solution and ketoconazole cream with improvement in their nail discoloration after two months. We introduce a term that specifically describes fungal etiology-associated green nail syndrome: viridionychia.

## Introduction

Green nail syndrome is a descriptive term for fingernails or toenails that are partially or completely green in appearance. It was originally described in 1944 as Goldman-Fox syndrome in association with *Pseudomonas aeruginosa* infection of the nail plate. However, green nail syndrome associated with bacterial infection is more generally referred to as chloronychia [1].

*Candida parapsilosis* is a fungal organism that has also been associated with green nail discoloration. It has been identified as an opportunistic pathogen in patients with systemic disease or onycholysis. The nail plate discoloration caused by *Candida parapsilosis* can be diffusely green or black or a linear streak of black, green, white, or yellow [2,3].

Two women with green nail syndrome associated with *Candida parapsilosis* are described, and the potential etiologies of green nail syndrome are reviewed. The term chloronychia is firmly established to refer to green nail discoloration associated with a bacterial infection. We suggest that onychomycosis with concomitant green nail discoloration be referred to as viridionychia.

## Case presentation

Case 1

A 70-year-old woman presented for evaluation and management of an asymptomatic green discoloration of her left fourth fingernail of six months duration. Her medical history was significant for chronic back pain, gastroesophageal reflux disease, hypertension, and overactive bladder. She was being treated with tramadol, omeprazole, losartan, and tolterodine, respectively.

The patient’s discolored nail plate had been treated by her primary care physician. Twice daily triamcinolone acetonide 0.025% cream was applied to the proximal nail fold of her left fourth fingernail. There was no improvement after one month of therapy.

**Figure 1:**
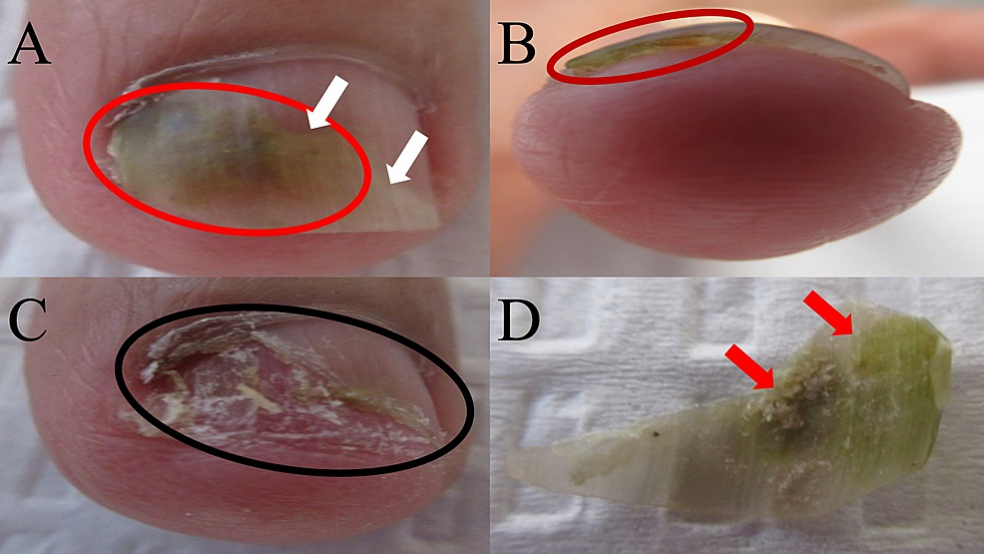
Fungal viridionychia secondary to Candida parapsilosis presenting as green nail syndrome of the left fourth fingernail. A 70-year-old woman presented with green discoloration (red oval) of the left fourth fingernail (A); there was also distal onycholysis (detachment) of the nail plate (white arrows show the area located at the distal portion of the attached nail plate and proximal portion of the detached nail plate). Green discoloration (red oval) was notable when viewing the inferior surface of the onycholytic nail plate (B). The distal nail bed of the fourth fingernail (C) was exposed after partial avulsion of the discolored portion of the distal nail plate (black oval). The inferior surface of the removed nail plate (D) shows diffuse green pigmentation (red arrows).

Clinical examination of her left fourth fingernail was performed. It showed diffuse green discoloration of the nail plate extending from the proximal nail fold. There was also distal onycholysis of her nail plate (Figure 1).

The distal detached left fourth fingernail was removed for bacterial culture, fungal culture, and pathology evaluation. The exposed nail bed was treated topically. Twice daily she applied clindamycin 1% solution (which was used as a desiccant because the antibiotic is dissolved in alcohol) and then ketoconazole 2% cream.

The biopsy of the affected nail was stained with not only hematoxylin and eosin (H&E) stains but also periodic acid-Schiff (PAS) stain. Fungal organisms (pseudohyphae and scattered yeast forms) were observed on the PAS-stained sections. The bacterial culture did not grow any organisms; however, the fungal culture grew *Candida parapsilosis* after four weeks.

Itraconazole is a selective inhibitor of cytochrome P450, an enzyme involved in the metabolism of many medications. Due to the potential risk of interaction between her current medications (losartan, omeprazole, and tolterodine) with oral antifungal agent itraconazole, she continued twice daily topical treatment with clindamycin 1% solution followed by ketoconazole 2% cream. She continued to remove this distal non-attached nail plate with nail clippers every other week. The green discoloration did not recur, and the distal onycholysis was resolving at the two-month follow-up visit.

Case 2

A 70-year-old woman presented with green discoloration of her right thumbnail of five months duration. Her history revealed hyperlipidemia for which she was taking atorvastatin. Three months prior to the onset of her green thumbnail, she had seen her primary care physician who noted the nail had red-purple discoloration and was tender, suggestive of a subungual hematoma.

**Figure 2:**
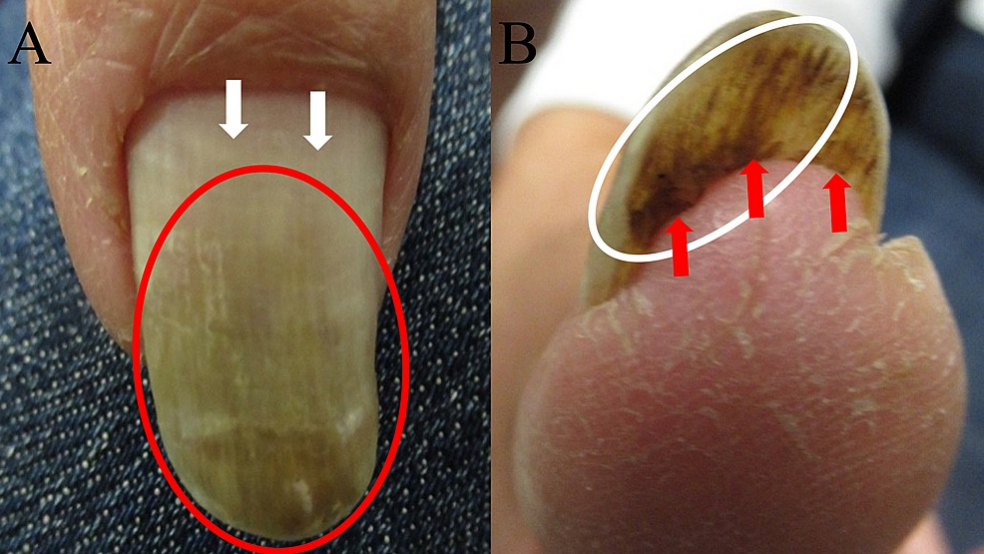
Candida parapsilosis viridionychia presenting as green dyschromia of the right thumbnail. A 70-year-old woman presented with green discoloration (red oval) of her right thumbnail (A); there was distal detachment (onycholysis) of nearly the entire nail plate from the distal edge of the lunula (white arrows) to the tip of the nail. A view of the inferior surface of the onycholytic nail plate (white oval) of the right thumbnail (B) shows green discoloration (red arrows).

Clinical examination of her right thumbnail showed a thickened nail plate with distal green discoloration; there was also extensive distal onycholysis of the nail plate (Figure 2). The detached portion of the thumbnail was removed for bacterial culture, fungal culture, and pathology evaluation. She was started on twice daily application of topical clindamycin 1% solution followed by mupirocin 2% ointment to her right thumb nail bed.

Microscopic examination of the nail plate showed PAS-positive pseudohyphae and yeast. The bacterial culture did not yield any organisms. After four weeks, the fungal culture grew *Candida parapsilosis*, confirming the diagnosis of onychomycosis with green nail plate discoloration.

Cytochrome P450 is an enzyme involved in the metabolism of many medications, and itraconazole is a selective inhibitor of this enzyme. Therefore, oral antifungal medication itraconazole was not recommended due to the potential risk of interaction with the atorvastatin she was receiving. Topical therapy was continued with twice daily application of clindamycin 1% solution (used as a desiccant because of its alcohol base) and ketoconazole 2% cream along with regular nail clipping. Improvement in her green nail plate discoloration was seen at the two-month follow-up visit, and she plans to continue treatment until her normal-appearing new nail has completely grown.

## Discussion

Chromonychia refers to nail plate discoloration. Green nail syndrome, presenting as green discoloration of the nail plate, is usually reported in association with *Pseudomonas aeruginosa*. It is frequently a result of colonization of the organism and rarely an active infection of the pathogen [1].

Green nail syndrome frequently only affects a single digit. The characteristic green chromonychia of the nail may be monochromatic or accompanied by other nail plate discoloration such as yellow or brown. In addition to subungual hyperkeratosis, the surface of the nail plate may be altered and/or white streaks may be present [4].

A recent study demonstrated that green nail syndrome was identified concurrently with onycholysis in 87% of individuals, as it was in both of our patients. Distal onycholysis is most commonly observed. The detached nail plate may provide an opportunity for moisture to accumulate, thereby fostering colonization of bacterial and fungal organisms [4].

Green nail syndrome can be associated with either a singular bacterial infection, a singular fungal infection, or a polymicrobial infection [3-12]. Single bacterial etiologies for green nail syndrome are caused by gram-negative organisms. They include *Citrobacter braakii*, *Pseudomonas aeruginosa*, and *Pseudomonas oryzihabitans* (Table 1) [5-7].

**Table 1:** Single bacterial etiologies of green nail discoloration Abbreviations: A, age (years); M, man; Ref, references; S, sex; W, woman ^a^Initial treatment with amoxicillin/clavulanic acid was unsuccessful [5]. ^b^Also referred to as pseudo pseudo-Hutchinson’s sign [6].

Bacteria	A	S	Digit	Clinical presentation	Diagnosis	Treatment	Ref
Citrobacter braakii	34	W	Right index finger	Green discoloration of the entire nail plate, distal onycholysis, and an abscess at the proximal nail fold for one month	Bacterial culture grew *Citrobacter braakii*	Complete nail avulsion, drainage of the abscess, and ciprofloxacin led to no recurrence of the symptoms at five day follow up^a^	[5]
Pseudomonas aeruginosa	75	M	Right great toe	Distal onycholysis and green subungual discoloration of the toenail into the proximal nail fold^b^ for six months	Bacterial culture grew *Pseudomonas aeruginosa*	Twice daily application of ofloxacin otic 0.3% solution for six months led to resolution	[6]
Pseudomonas oryzihabitans	24	W	Left third finger	Asymptomatic yellow-green discoloration of the nail adjacent to the lateral nail fold for four days	Bacterial culture grew *Pseudomonas oryzihabitans*	Topical nadifloxacin cream for two months resolved the discoloration	[7]

*Citrobacter braakii* is a gram-negative bacillus that can cause bacteremia and sepsis in immunocompromised patients. However, it has been associated with chloronychia in a 34-year-old woman who presented with painful swelling around her right index fingernail one month after injury to this digit. Two weeks prior to her presentation, she noticed a green hue to her nail plate, which progressively worsened; she was empirically treated with amoxicillin/clavulanic acid which did not improve her symptoms [5].

Clinical examination revealed a grossly green nail plate with distal onycholysis and abscess at the proximal nail fold. Bacterial culture after onychectomy and drainage of the abscess grew *Citrobacter braakii*. The patient was treated with ciprofloxacin; all symptoms had resolved, without recurrence, at the five-day follow-up [5].

*Pseudomonas aeruginosa* is a gram-negative coccobacillus with a predilection for moist, aquatic environments. It produces pyocyanin and pyoverdine, which are pigmented virulence factors that give its characteristic blue-green color in green nail syndrome. When *Pseudomonas aeruginosa* is associated with green nail discoloration, it is usually described as Goldman-Fox syndrome [1].

*Pseudomonas aeruginosa* is an opportunistic pathogen. It usually infects individuals with preexisting or concurrent nail dystrophy such as onycholysis, paronychia, or psoriasis. However, individuals may also acquire Goldman-Fox syndrome from constant water exposure or from working in a healthcare facility [1].

A 75-year-old man with no history of nail trauma presented with green subungual discoloration of his right great toenail extending to and involving his proximal nail fold (pseudo pseudo-Hutchinson’s sign) and distal onycholysis for six months. A partial avulsion of onycholytic nail was performed; microscopic examination of the nail plate after gram stain found subungual bacteria, and bacterial culture of the nail plate grew *Pseudomonas aeruginosa*. Topical treatment with oflaxacin otic 0.3% solution twice daily led to its resolution with no recurrence of discoloration after six months [6].

*Pseudomonas oryzihabitans* is a gram-negative bacillus that has been associated with yellow-green discoloration of the nail plate in one patient. This opportunistic pathogen can also cause bacteremia, peritonitis, and sepsis. In contrast to *Pseudomonas aeruginosa*-induced chloronychia, the unique yellow-green hue of *Pseudomonas oryzihabitans *can be a distinguishing clinical presentation of nail dyschromia from this organism [7].

A 24-year-old woman presented with yellow-green discoloration of her lateral left third fingernail for four days after removing her gel nail polish. Bacterial culture of the nail plate grew ciprofloxacin-susceptible *Pseudomonas oryzihabitans*. Treatment with topical nadifloxacin cream led to complete resolution of the nail plate discoloration after two months [7].

Currently, to the best of our knowledge, only *Candida* species have been described as the sole fungal pathogen in patients - including the women in this report - with green nail syndrome (Table 2) [3,8]. The specific etiology of the green nail plate dyschromia remains to be determined. *Candida albicans* is a species that commonly causes genital, mucocutaneous, and urinary tract infections, particularly in immunocompromised hosts.

**Table 2:** Single fungal etiologies of green nail discoloration Abbreviations: A, age (years); C1, case 1; C2, case 2; CR, current report; M, man; mg, milligrams; PCR, polymerase chain reaction; Ref, references; S, sex; soln, solution; W, woman ^a^Clindamycin solution was used for its alcohol base as a desiccant, not its antibiotic properties. ^b^This initial presentation was suggestive of a subungual hematoma.

Fungus	A	S	Digit	Clinical presentation	Diagnosis	Treatment	Ref
Candida albicans	33	W	Multiple fingers	Light and dark green discoloration of multiple nails with subungual caseous material for one month	Fungal culture grew *Candida albicans*	Not described	[8], C2
Candida parapsilosis	38	W	Right index finger	Cycles of green-black discoloration and onycholysis of the nail every two to three years	Fungal culture and PCR sequencing analysis found *Candida parapsilosis*	Five cycles of oral itraconazole 200 mg twice daily for one week then three weeks without therapy led to complete resolution of onychomycosis	[3]
Candida parapsilosis	70	W	Left fourth finger	Green-brown discoloration and distal onycholysis of the nail	Fungal culture grew *Candida parapsilosis*	Clindamycin 1% soln^a^ and ketoconazole 2% cream twice daily led to improvement of the discoloration after two months	CR, C1
Candida parapsilosis	70	W	Right thumb	Thickened nail and distal green discoloration of the nail with onycholysis; three months prior, the nail was red-purple and tender^b^	Fungal culture grew *Candida parapsilosis*	Clindamycin soln^a^ and ketoconazole 2% cream twice daily led to improvement of the discoloration after two months	CR, C2

A 33-year-old woman presented with different shades of green discoloration of multiple nails. She attributed this discoloration to hand washing her children’s diapers because her mother had similar symptoms after washing their diapers as well. Microscopic examination of subungual scrapings found yeast-like organisms, and culture of the caseous material under her nail plates grew *Candida albicans* [8].

*Candida parapsilosis* is a species that has recently been identified as pathogenic, particularly in patients receiving chemotherapy or hemodialysis treatment and in patients with indwelling devices, low birth weight, neutropenia, premature birth, and ventricular septal defect [9]. In addition, it is commonly isolated from the hands [9]. *Candida parapsilosis* has been described as the pathogen of green nail syndrome in three individuals, including our own patients [3].

A 38-year-old woman with history of nail injury and detachment 12 years earlier presented with recurrent cycles of green-black discoloration and onycholysis of her right first fingernail every two to three years. Microscopic examination after potassium hydroxide preparation revealed spores, and polymerase chain reaction of the cultured strain with gene analysis identified *Candida parapsilosis*. She was effectively treated with five monthly cycles of therapy (one week of oral itraconazole 200 mg twice daily then three weeks without treatment) with no recurrence of her symptoms [3].

Polymicrobial infections of the nail have also been observed in individuals with green nail syndrome (Table 3) [8,10-12]. In one report of 23 patients with green nail syndrome, fungal and bacterial co-infection was found in 65% of the patients [13]. Although co-infection was identified, we suspect the green nail discoloration was secondary to the virulent pigments (pyocyanin and pyoverdine) produced by *Pseudomonas aeruginosa*.

**Table 3:** Polymicrobial etiologies of green nail discoloration Abbreviations: A, age (years); C1, case 1; C2, case 2; C3, case 3; M, man; mg, milligrams; PCR, polymerase chain reaction; Ref, references; S, sex; W, woman ^a^1:1000 in 70% alcohol [8]. ^b^2% iodine crystals dissolved in benzene [8]. ^c^Pulsed fluconazole therapy (150 mg weekly) for one year trialed but not effective [11]. ^d^Prior to this, she was treated with systemic antibiotics for one week without resolution of her symptoms [11].

Microbes	A	S	Digit	Clinical presentation	Diagnosis	Treatment	Ref
*Candida albicans* and *Pseudomonas aeruginosa*	51	W	Left thumb	Thickened, hyperkeratotic, striated, and dark green discoloration of the nail for two months	Cultures grew *Candida albicans* and *Pseudomonas aeruginosa*	Not described	[8], C3
*Candida albicans* and *Pseudomonas aeruginosa*	40	W	Third finger	Brown-green discoloration of the nail adjacent to the lateral nail fold for a few months	Microbiological examination found *Candida albicans* and *Pseudomonas aeruginosa* co-infection	Oral fluconazole 200 mg per week, topical ciprofloxacin eye drops, and topical ciclopirox with near complete resolution of the discoloration after three months	[10]
*Candida tropicalis* and *Pseudomonas aeruginosa*	50	W	Right thumb	Distal blue-green discoloration, distal onycholysis, and lateral and longitudinal nail ridging of the nail with subungual caseous material	Cultures grew *Candida tropicalis* and *Pseudomonas aeruginosa*	Soaking the nail in mercury bichloride solution^a^ twice daily and topical application of 2% iodine^b^ led to improvement in the nail discoloration	[8], C1
*Fusarium solahi* and *Pseudomonas aeruginosa*	63	W	Left thumb	Onycholysis, thickening, and green discoloration of half the nail for at least three months	Cultures and PCR sequence analysis found *Fusarium solahi* and *Pseudomonas aeruginosa*	Fluconazole was not effective^c^; itraconazole 200 mg and ciprofloxacin 500 mg daily led to resolution of the green discoloration after three months	[11], C1
*Fusarium solahi* and *Pseudomonas aeruginosa*	74	W	Right thumb	Progressive pain and onycholysis, periungual desquamation, and yellow-green discoloration of the thumbnail for one year	Cultures and PCR sequence analysis identified *Fusarium solahi* and *Pseudomonas aeruginosa*	Oral antibiotics^d^ followed by terbinafine 250 mg daily and ciprofloxacin 500 mg daily for two months led to resolution of the dyschromia	[11], C2
*Klebsiella pneumoniae* and *Pseudomonas aeruginosa*	53	W	Both thumbs	Slowly progressive green discoloration and psoriasis-induced onycholysis of both thumbnails	Bacterial culture found *Klebsiella pneumoniae* and *Pseudomonas aeruginosa*	Discoloration resolved after two weeks of treatment with topical nadifloxacin	[12]

A 51-year-old woman who worked as a hand sewer presented with thickened, hyperkeratotic, and dark green discoloration of her left thumbnail of two months duration. Fungal and bacterial smears of the nail showed yeast and bacilli. Fungal and bacterial cultures of the nail grew *Candida albicans* and *Pseudomonas aeruginosa*, confirming a co-infection [8].

A 40-year-old woman presented with green-brown discoloration of her third fingernail adjacent to the lateral nail fold of a few months duration. Microbiological examination identified the presence of both *Candida albicans* and *Pseudomonas aeruginosa*. Treatment with oral fluconazole 200 mg weekly, topical ciprofloxacin eye drops, and topical ciclopirox for three months led to resolution of the green-brown discoloration [10].

A 50-year-old woman presented with a blue-green distal discoloration of her right thumbnail and associated subungual caseous material of six months duration following an injury to the digit. Potassium hydroxide staining of the caseous material found yeast-like cells and bacilli, and cultures grew *Candida tropicalis* and *Pseudomonas aeruginosa*. She was successfully treated by soaking her thumbnail in an alcohol-based mercury bichloride solution twice daily followed by the topical application of iodine crystals 2% in benzene [8].

*Fusarium* species are opportunistic fungal pathogens most commonly found in soil. In immunocompetent patients, they may be associated with onychomycosis, especially in toenails, and keratitis. In immunocompromised patients, *Fusarium* infections are often disseminated [11].

A 63-year-old woman presented with green discoloration, onycholysis, and thickening of her left thumbnail. Although the onycholysis began after damaging this nail two years prior, the chromonychia only appeared in the preceding three months. She was treated with pulse fluconazole 150 mg therapy weekly for one year without resolution of her symptoms [11].

A potassium hydroxide preparation of the subungual green scrapings found fungal elements; polymerase chain reaction sequence analysis of the material identified the pathogen as *Fusarium solani*. Bacterial culture of the same green material grew *Pseudomonas aeruginosa*. She was concurrently treated with itraconazole 200 mg daily and ciprofloxacin 500 mg daily for three months which resolved the green dyschromia but left a residual yellow nail discoloration [11].

A 74-year-old woman presented with progressive onycholysis, pain, and yellow-green discoloration of her right thumbnail for one year after she started working in a restaurant kitchen. Pustules and discharge had also been present for one week. However, her symptoms and findings were not responsive to a week-long course of systemic antibiotics [11].

Polymerase chain reaction sequence analysis of the subungual nail scrapings revealed 100% similarity with *Fusarium solani*. Bacterial cultures of the nail grew *Pseudomonas aeruginosa*. She was concurrently treated with terbinafine 250 mg daily and ciprofloxacin 500 mg daily for two months which cleared the green dyschromia but did not resolve her yellow discoloration and onycholysis [11].

*Klebsiella pneumoniae* is a gram-negative bacillus commonly associated with hospital-acquired infections [14]. This opportunistic pathogen has been described with bacteremia, pneumonia, and urinary tract infections [14]. *Klebsiella pneumonia*, in association with *Pseudomonas aeruginosa*, has been identified as a pathogen in one patient with green nail discoloration [12].

A 53-year-old woman with history of psoriasis-induced onycholysis (being treated with efalizumab) presented with worsening green discoloration of both thumbnails of several weeks duration. Bacterial culture of the nail scrapings grew both *Klebsiella pneumoniae* and *Pseudomonas aeruginosa*. Treatment with topical nadifloxacin normalized her nail color [12].

Green nail syndrome is often a result of colonization of organisms rather than an infection. Treatment generally involves removal of the detached nail, which can be used for bacterial culture, fungal culture, and staining of the nail plate, in addition to desiccation of the nail bed and/or application of a topical antibacterial or antifungal agent. If persistent, recurrent, affecting multiple digits, or affecting more than half of the nail plate, systemic agents can also be used [15].

*Pseudomonas*-associated green nail syndrome can be treated with topical agents including acetic acid 1% compresses, bacitracin, gentamicin 0.3% eye drops, and sodium hypochlorite 2% solution. In some patients, oral antibiotics to which *Pseudomonas aeruginosa* is susceptible - such as quinolones - may be used. The patients we discussed had resolution of their chloronychia with oral ciprofloxacin, topical nadifloxacin, or topical ofloxacin [1,5-7,15].

In one report, a 35-year-old man presented with distal onycholysis and associated green discoloration of his left thumbnail for over one year. He failed treatment with oral itraconazole 200 mg and levofloxacin 200 mg daily, but bacterial culture and sensitivity testing identified growth of tobramycin-sensitive *Pseudomonas aeruginosa*. He was successfully treated with topical tobramycin 3 mg/ml eye drops applied twice daily for three weeks [16].

Onychomycosis can be treated with oral or topical antifungal agents depending on the clinical circumstance. Topical antifungal agents can be used for distal onychomycosis affecting less than half of the nail plate, onychomycosis affecting up to three or four nails, and superficial onychomycosis. Daily application of ciclopirox 8% lacquer to the nail plate, hyponychium, and surrounding skin for 24 weeks for fingernails (48 weeks for toenails) has a 29-36% mycologic cure rate. Topical daily application of efinaconazole 10% solution or tavaborole 5% solution to the nail plate and its undersurface, hyponychium, and nail folds for 48 weeks has a 53-55% and 31-35% mycologic cure rate, respectively [17,18].

Alternatively, oral therapy may be indicated in individuals with distal onychomycosis affecting more than half of the nail, onychomycosis involving more than three or four nails, and proximal subungual onychomycosis. If a dermatophyte is suspected, treatment with oral terbinafine 250 mg for six weeks is recommended for fingernails (12 weeks for toenails) with a 70-79% cure rate. Oral itraconazole 200 mg twice daily for one week per month for two months is effective in fingernail-associated onychomycosis (three months of treatment for toenails) with a 54-61% cure rate [17,18].

Nonprescription agents (such as topical mentholated ointment, tea tree oil, and snakeroot extract) and laser therapy have been investigated in the treatment of onychomycosis. Of these, mycotic cure rates were variable: between 28 and 61%. However, none have been described specifically in the treatment of onychomycosis-associated green nail discoloration. Neodymium:yttrium-aluminum-garnet laser therapy one to three times, each four to eight weeks apart, had the highest cure rate (61%) [18].

The etiology of a green nail cannot be determined by the clinical presentation alone. It is most commonly caused by a *Pseudomonas *species, a bacterial organism. However, it can also occur as a result of a fungal organism such as a *Candida* species or the presence of both bacterial and fungal organisms. Therefore, culture - for both bacteria and fungi - of the onycholytic green nail plate that has been removed may be helpful in selecting the appropriate antibacterial drug, antifungal agent, or both [3-12].

The patients with fungal-associated chromonychia, who were reviewed in this paper, had resolution of their discoloration with treatment of the pathogen. The individuals with solely fungal etiologies found improvement with topical 2% ketoconazole cream and a desiccant or with oral itraconazole. The individuals with concurrent bacterial and fungal-associated green nail syndrome had resolution of their green discoloration (likely due to treatment of *Pseudomonas aeruginosa*) with a combination of an oral or topical quinolone (ciprofloxacin or nadifloxacin) and oral fluconazole plus topical ciclopirox, oral itraconazole, or oral terbinafine [8,10-11].

When treating fungal infections, it is important to consider the potential drug interactions associated with oral antifungal medications such as itraconazole; the drug may interact with agents that are metabolized through the cytochrome P450 pathway. Therefore, it may be prudent to evaluate the patient’s current medications for potential drug interactions before initiating treatment with a systemic antifungal drug. In our patients, we opted for topical antifungal treatment which was successful in resolving their discoloration.

In summary, a bacterial etiology for green nail syndrome is more commonly observed. It is usually associated with *Pseudomonas aeruginosa*. In this setting, the nail condition is typically termed chloronychia or Goldman-Fox syndrome.

To the best of our knowledge, a paucity of patients with fungal-associated green nail syndrome have been described. Thus far, only *Candida* species have been reported as pathogens. However, onychomycosis with concurrent green discoloration of the nail plate may be more common than published literature suggests.

We propose viridionychia as the descriptive term for onychomycosis with concurrent green nail discoloration. Viridionychia is based on the Latin translation of the color green - viridis. The terminology fungal viridionychia may be useful to differentiate patients with this unique etiology of green nail syndrome from the individuals with bacterial chloronychia.

## Conclusions

Green nail syndrome can be broadly used to describe green discoloration of the nail plate. Although a bacterial etiology is most common, fungal and polymicrobial infections have been identified through fungal cultures of nails from individuals with green nail syndrome. Both of our patients had green discoloration of their nails in association with *Candida parapsilosis* which were successfully treated with a topical alcohol solution and ketoconazole cream. Identifying the correct etiology of green nail syndrome is imperative to appropriately treat the patient’s nail discoloration. We propose the term viridionychia to be used when specifically describing individuals who have a fungal etiology for their green nail syndrome.
